# Clinicopathological characteristics of pulmonary epithelioid hemangioendothelioma: A report of four cases and review of the literature

**DOI:** 10.3892/ol.2014.2566

**Published:** 2014-09-26

**Authors:** JINCHEN SHAO, JIE ZHANG

**Affiliations:** Department of Pathology, Shanghai Chest Hospital, Shanghai Jiao Tong University, Shanghai 200030, P.R. China

**Keywords:** clinicopathological characteristics, pulmonary epithelioid hemangioendothelioma, low-grade malignant vascular tumor

## Abstract

This study aimed to investigate the clinicopathological characteristics, differential diagnosis and prognosis of pulmonary epithelioid hemangioendothelioma (PEH). PEH is a rare low-grade malignant vascular tumor. The cause of PEH remains unclear. Patient prognosis is unpredictable, with life expectancy ranging from 1 to 15 years due to the fact that estrogenic receptors behave inconsistently within the tumor and the occurence of the disease in male patients does not support the usual hormonal hypothesis. The clinical manifestations, imaging findings, histopathological characteristics and immunohistochemical phenotypes of four cases of epithelioid hemangioendothelioma occurring in the lung were retrospectively analyzed, and a review of the associated literature was conducted. The age of onset for the four PEH cases was 25–54 years, and the disease manifested as multiple nodules in the lungs or pleura. All of the patients underwent lobectomy or pulmonary wedge resection. The morphology of the tumor cells was epithelioid or spindle-shaped with abundant eosinophilic cytoplasm in which lumina or vacuoles containing erythrocytes were observed. The cells were arranged in nests and cords with degenerated interstitial mucoid. The morphology of the majority of the tumor cells was moderate, including mild atypia and little mitosis or necrosis. Immunohistochemical staining showed positive results for CD31, CD34 and F8. PEH is a rare low- to moderate-level tumor occurring in the lungs with differentiation toward vascular endothelial cells. Clinically, it is difficult to distinguish from a variety of other benign and malignant lung diseases. For diagnosis, a distinction must be made from other diseases such as chronic granulomatous disease, amyloid nodules, hamartoma, primary and metastatic lung cancers, malignant mesothelioma and vascular sarcoma. In the present study, the clinicopathological features of four cases of PEH were investigated and the associated literature was reviewed. The results of this study may improve understanding with regards to the diagnosis and therapeutic options for patients with PEH.

## Introduction

Pulmonary epithelioid hemangioendothelioma (PEH) is the current term for a rare neoplasm originally described by Dail and Liebow in 1975 as intravascular sclerosing bronchioalveolar tumor (IVBAT) of the lung (1,2). PEH is a rare pulmonary neoplasm of vascular origin with fewer than 50 cases reported in the literature (3–6). PEH typically manifests as multiple bilateral lung nodules that are usually discovered incidentally in young or middle-aged Caucasian women, although cases in children and the elderly have also been reported (4,7). Male, symptomatic, the presence of cough, hemoptysis, chest pain, multiple unilateral nodules, pleural effusion, metastases to more than one site and lymph node metastases are the factors associated with a poor prognosis. Symptomatic patients and the presence of a pleural effusion are independent predictors of survival in patients with PEH (7). Epithelial hemangioendothelioma has also been reported to originate in the liver, head and neck area, oral mucosa, bone, mediastinum, diaphragm and brain (8,9).

PEH is a rare low-grade malignant vascular tumor that occurs in the lungs and, due to its rarity, it is easy to clinically misdiagnose PEH as other lung diseases. In the present study, four cases of PEH, which were diagnosed and treated at Shanghai Chest Hospital, Shanghai Jiao Tong University (Shanghai, China), were observed and analyzed with respect to the clinical manifestations, imaging findings, histopathological characteristics, immunohistochemical phenotypes and prognosis. This study was performed according to the Declaration of Helsinki and was approved the the ethics committee of Shanghai Chest Hospital, Shanghai Jiao Tong University (Shanghai, China). Written informed consent was obtained from all patients.

## Case reports

### Clinical manifestations

**Figure 1:**
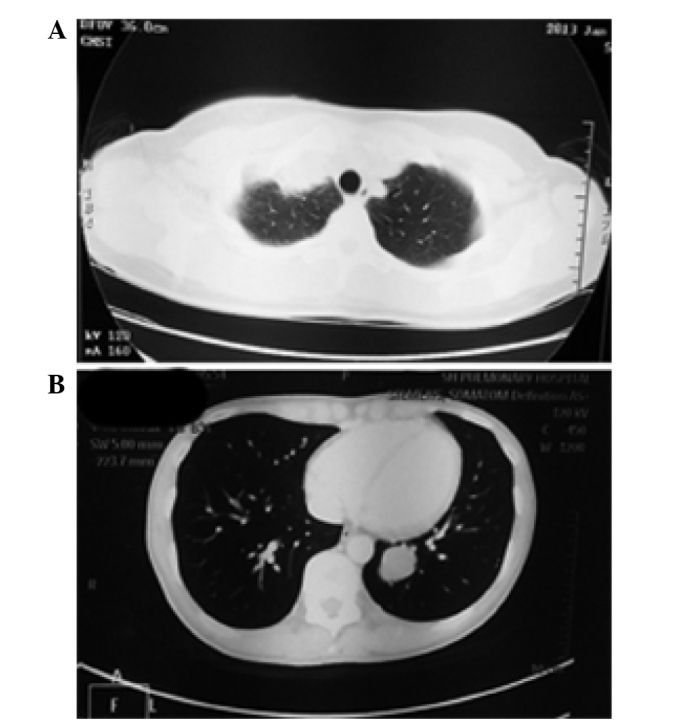
(A) Case 3: A 46-year-old female with no obvious symptoms. Irregular clumps were found below the pleura of the right upper lung on a chest CT during a physical examination. (B) Case 4: A 30-year-old female with no obvious symptoms. Multiple nodules in both lungs were revealed on a chest CT during a physical examination. CT, computed tomography.

The present study describes four cases of PEH that were diagnosed at the Chest Hospital Affiliated to Shanghai Jiaotong University from 2006 to 2013. Two of the cases were surgical patients at the hospital, while the other two cases were consultation patients from other hospitals. Case 1 was a 54-year-old male with multiple lung nodules that were revealed during a preoperative examination for cholecystitis. Case 2 was a 54-year-old female who was admitted for chest tightness and fatigue, whereby X-ray examination revealed a right pleural effusion. Case 3 was a 46-year-old female with no obvious symptoms; yet irregular clumps were found below the pleura of the right upper lung on a chest computed tomography (CT) during a routine physical examination (Fig. 1A). Case 4 was a 30-year-old female who was also without obvious symptoms, and multiple nodules in both lungs were revealed on a chest CT during a routine physical examination (Fig. 1B). Cases 3 and 4 underwent lobectomy, while cases 1 and 2 underwent pulmonary wedge resection. A seven-year postoperative follow-up for case 1 showed that the patient’s condition was stable, with no significant progression. Persistent pleural effusion was observed in case 2, and the patient received chemotherapy at another hospital, but succumbed to the disease after three years. Cases 3 and 4 were six and five months into the postoperative follow-up period at the time of writing, respectively. (Table I)

### Materials and methods

All of the specimens were fixed in 4% neutral formalin, embedded in paraffin and stained with hematoxylin and eosin. Briefly, following deparaffinization, rehydration, heat-induced epitope retrieval and endogenous peroxidase blocking, the slides were incubated with primary antibodies for 1 h. The primary monoclonal antibodies, including mouse anti-human cluster of differentiation 31 (CD31) (1:200 dilution), mouse anti-human CD34 (1:200 dilution, mouse anti-human creatine kinase (CK) (1:100 dilution), mouse anti-human CK7 (1:200 dilution), mouse anti-epithelial membrane antigen (EMA) (1:200 dilution), mouse anti-human calretinin (1: 200 dilution), mouse anti-human desmin (1:200 dilution), mouse anti-human thyroid transcription factor 1 (TTF1) (1:200 dilution) and mouse anti-human vimentin (1:200 dilution) and polyclonal rabbit anti-human factor VIII (F8) (1:200 dilution) were all purchased from Dako (Carpinteria, CA, USA). The specimens were subsequently subjected to the polyclonal goat anti-rabbit anti-mouse secondary antibody (Dako) (dilution 1:500) for 30 min and visualized using 3,3′-diaminobenzidine tetrahydrochloride as chromogen using an Envision system (Dako) (10).

### Case 1

Two specimens from the pulmonary wedge resection of the left upper lobe were submitted for examination. One gray nodule was observed in each section, with diameters of 0.6 and 1 cm. Two other specimens from the pulmonary wedge resection of the left lower lobe were submitted for examination. One gray nodule was observed in each specimen, with diameters of 0.5 and 0.9 cm.

### Case 2

Two specimens from the pulmonary wedge resection of the right upper lobe were submitted for examination. Two patchy thickenings were found on the pleural surface, and the sections were gray and hard, measuring 3×2×1 cm and 1.5×1×0.5 cm.

### Case 3

The right upper lobe specimen was submitted for examination. A lump 3×2×2.1 cm in size was found in the upper right tip segment that was pale yellow-gray in color, hard, and invading into the pleura with pleural adhesions and thickening. Multiple gray nodules were also found in the middle and lower segments of the examined right lung lobe and right pleura, 0.3–0.5 cm in diameter.

### Case 4

A specimen of the left lower lobe was submitted for examination. A lump 3×3×2.5 cm in size was found in the basal segment of the left lower lobe that was gray-yellow, hard, well-defined and located 0.5 cm from the pleura. At 2 cm distant from the lump in the basal segment, another lump 1.5×1.5×1.4 cm in size was observed that was also gray-yellow, hard, well-defined and located 1 cm from the pleura.

### Microscopy

**Figure 2:**
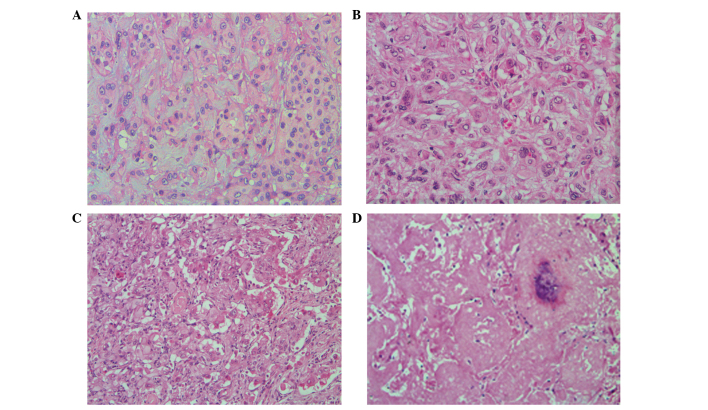
Histological examination characteristics of pulmonary epithelioid hemangioendothelioma (hematoxylin and eosin staining) (A, case 1; B, case 2; C, case 3; D, case 4). (A) Neoplasms are composed of short cords and nested tumor cells, and interstitial mucus degeneration (magnification, ×200). (B) Typically, the lumen or cavity in the tumor cytoplasm contain single or multiple erythrocytes (magnification, ×200). (C) Tumor cells are rich in certain areas, with marked atypia. The tumor cells are arranged in solid nests and duct-like structures, and form papillary structures in the blood vessels (magnification, ×200). (D) Tumor cells show multiple small nodules and local cerebral calcification at the necrotic center (magnification, ×100).

The tumors were composed of the tumor cells arranged in short cords and nests with degenerated stromal mucoid. The tumor cells were medium in size, polygonal or spindle shaped, with unclear cell boundaries. The cytoplasm was abundant and eosinophilic, and the nuclei were round, with small nucleoli showing mild or moderate atypia. The lumen or vacuolization containing one or more erythrocytes was commonly observed in the cytoplasm of the tumor cells (Fig. 2A and B). In case 4, the tumor cells were abundant in some areas, with obvious atypia; the tumor cells were arranged in solid nests and pseudoglandular structures, growing toward the surrounding alveoli and filling the alveolar space. The tumor cells in other areas formed papillary structures in the blood vessels (Fig. 2C). In case 3, the tumor cells were growing in multiple small nodules, with abundant cells surrounding the nodules and sclerosis at the nodular centers that was similar to hyaline degeneration or necrosis, and calcifications were observed in the necrotic center (Fig. 2D).

### Immunohistochemistry

**Figure 3:**
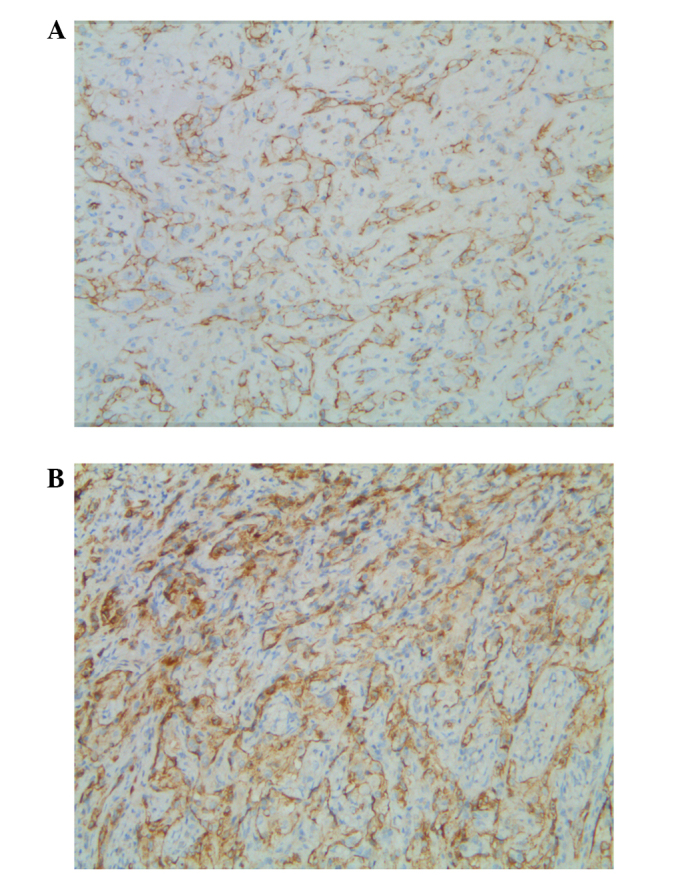
Immunohistochemical characteristics of epithelioid hemangioendothelioma (Envision; magnification, ×100) (A, case 1; B, case 3). Expression of (A) cluster of differentiation 31 and (B) factor VIII in the tumor cells.

The tumor cells of all four cases were positive for CD31, F8 and vimentin; CD34 expression was positive in three cases; and EMA was focally positive in one case. The tumor cells of all four cases were negative for other markers, including TTF1, CK, CK7, calretinin and desmin (Fig. 3A and B). The pathological diagnosis was PEH in each case.

## Discussion

Epithelioid hemangioendothelioma is a low-grade malignant vascular tumor that was previously considered an intermediate vascular tumor, but was reclassified as a low-grade angiosarcoma in the 2002 WHO classification (11). It often occurs in the superficial and deep soft tissues of the extremities, and cases occurring in lungs are rare and often multifocal (2,12). PEH was first reported by Dail and Liebow (13).

PEH often occurs in middle-aged women, with a mean patient age of 40 years old. With no obvious symptoms, patients are often diagnosed on physical examination. For patients with symptoms, chest tightness, shortness of breath and difficulty breathing after exertion can occur; the tumors grow slowly, manifesting as chronic processes clinically, and pleural effusion is the first sign of pleural invasion (7,14–16). Among the four cases of PEH in this study, three cases were initially identified during physical examination and one case showed a right pleural effusion as the first sign.

The imaging of PEH is mainly characterized by multiple pulmonary nodules, rare solitary lesions and nodule diameters of 1–2 cm, although a diameter of >5 cm has also been reported (9,15). Calcifications and ossifications have occasionally been found in the lesions. If the lesions have invaded the pleura, changes such as pleural thickening and pleural effusion can occur (17). The four cases in this study all showed multiple lung nodules or lesions.

Most commonly, PEH presents as bilateral or unilateral multiple pulmonary nodules with clear boundaries, diameters of 0.3–2 cm, and pale gray or brown coloration. These can invade the pleura and cause pleural effusion or nodular thickening. Calcifications or ossifications can occur in the center of the nodules (18,19).

The intralesional vascular structure was unclear, and the tumors formed short, cord-like strips with solid nest structures by the eosinophilic endothelial cells, which were round or slightly fusiform. The stroma was light blue hyaline mucus with hyaline degeneration in certain cases. The tumor cells showed abundant, ill-defined cytoplasm and intracytoplasmic lumina or vacuoles, which at times contained single or multiple intraluminal red blood cells. These red blood cells were evidence of the differentiation of tumor cells into vascular endothelial cells. The tumor cells had mild to moderate atypia, with nuclear vacuolization, inconspicuous nucleoli and rare or missing mitoses. Some tumor nodule centers showed hardening and an acellular zone accompanied by coagulation necrosis, calcification and ossification. The abundant tumor cells surrounding the nodules broke into the alveolar cavity in papillary or polypoid structures. A number of the atypical morphologies were observed in approximately one-third of cases, which were manifested as the obvious atypia in tumor cells, including a mitotic count of >1/10 per high-powered field, fusiform cells and accompanying necrosis. These lesions were highly invasive, and tumors at this phase are malignant epithelioid hemangioendotheliomas, with a continuation of the morphology of epithelioid angiosarcoma (4,8,20,21).

PEH expresses a variety of vascular antigens, such as CD31, CD34, F8, friend leukemia integration 1 transcription factor, Ulex europaeus agglutinin type 1 and FKBNP12 (22,23). Among these, F8 was highly specific, but its sensitivity was the lowest; CD31 was relatively specific and highly sensitive, globally expressed in 90% of the cases; CK or EMA was focally expressed in 25–30% of the cases. In the present study, all four cases expressed CD31 and F8 (100%), three cases expressed CD34 (75%), one case was focally positive for EMA (25%) and no case expressed CK (0%).

Due to the fact that PEH is rare, clinically, it is easily confused with a variety of benign and malignant lung diseases (24). As PEH shows multiple lung nodules on imaging, it is often misdiagnosed as peripheral lung cancer with lung metastasis. The differential diagnosis of PEH includes chronic granulomatous disease, amyloid nodules, hamartoma, primary or metastatic lung cancer, malignant mesothelioma and angiosarcoma (25).

Chronic granulomatous diseases include tuberculosis, sarcoidosis and fungal granuloma. These diseases may present as multiple unilateral or bilateral pulmonary nodules on imaging. However, tuberculosis is positive in the clinical tuberculin test, and the effectiveness of anti-TB treatment can contribute to its identification. Among patients with active sarcoidosis, 80% show an increased level of angiotensin converting enzyme, and sarcoidosis is characterized by multiple mediastinal lymph nodes and hilar lymphadenopathy, with or without pulmonary nodular lesions. These are all pathologically expressed as epithelioid nodular hyperplasia. Sarcoidosis can show typical caseous necrosis, and sarcoidosis is characterized by concentric granulomas, implying that the outer layer arrangement of the collagen granuloma is onion-like, without necrosis or with only focal necrosis. Fungal granulomas show refraction spores inside and outside of multinucleated giant cells and histiocytes. Therefore, they may be clearly pathologically distinguished from PEH (26).

Patients with pulmonary amyloid nodules also show no obvious symptoms, and they are usually elderly patients with single or multiple pulmonary lesions found on physical examination, presenting similar clinical and radiological manifestations to PEH. Pathologically, nodular amyloidosis shows an irregular structure on microscopy, with homogeneous powder staining. Additionally, the nodules are surrounded by foreign body giant cells phagocytizing the amyloid, with occasional focal ossification and calcification. No tumor cells differentiated from mild or moderate atypical vascular endothelial cells, as in PEH, are observed. Congo red and methyl violet starch staining can confirm the diagnosis (27).

Chest imaging of hamartoma typically reveals a solitary and well-defined nodule, while multiple lesions are rare. Pathomorphologically, the nodule is mainly comprised of lobulated mature cartilage, and retraction of the bronchiolar epithelium may be observed. When a large mucus cartilage-like stroma of PEH appears, it must be distinguished from the true cartilage component of hamartoma. However, based on the findings of different imaging studies, its pathomorphology and immunohistochemical markers, it is not difficult to distinguish hamartoma from PEH.

The metastasis of primary lung adenocarcinoma within the lungs or the metastasis of extrapulmonary tumors to the lungs is the most common reason for the occurrence of multiple nodules in the lungs and, thus, it is not difficult to explain why PEH is often misdiagnosed as these tumor types. In addition, the nested and corded arrangement of the tumor cells and the myxoid stroma in PEH are also easily misdiagnosed as adenocarcinoma or mucinous adenocarcinoma on biopsy and during intraoperative freezing. However, the atypia of the tumor cells in primary or metastatic lung cancer are obvious, with clear mitoses. If the immunohistochemistry shows the expression of the epithelial cell marker and is negative for the vascular endothelial marker, such tumors can be differentiated from PEH.

In the case of PEH invading the pleura or a pleural effusion, PEH must be distinguished from malignant pleural mesothelioma. The tumor cells of the latter are characterized by bidirectional differentiation, with the expression of the epithelial markers CK and CK7, the mesenchymal marker vimentin, and mesothelial cell markers, such as calretinin, Wilms tumor 1, D2–40 and CK5/6. In addition, malignant pleural mesothelioma cells are negative for vascular endothelial marker (CD31 and CD34) expression.

Angiosarcoma is common in skin and rare in the lungs, and tends to present as a large solitary mass. Tumor vascular lumina, luminal lining epithelioid malignant cells, highly atypical tumor cells and mitoses are commonly observed under the microscope, and large lesions with spindle cells are also observable. Some PEHs may show certain atypical morphologies that are similar to epithelioid angiosarcoma, forming a morphological continuity with epithelioid angiosarcoma (28).

When the lesions of PEH inside one lung are relatively limited, surgery is the preferred treatment; however, when the lesions have invaded both lungs and cannot be completely resected, postoperative chemotherapy may be required, according to the condition of the patient. The average survival time of patients with asymptomatic pulmonary nodules is 15 years, and this can be >25 years in the best cases (12,13). Patients whose lung lesions were completely removed by surgery have shown long remissions or have been cured. Patients whose lesions were not completely removed and showed invasion into the airway, blood vessels and pleura showed poor prognosis; patients at advanced stages often died of respiratory failure (7,9). It has been reported that the survival rate for 40% of patients with PEH is less than five years (12,29). Since the prognosis for patients with PEH varies widely, long-term follow-up is necessary. For the four cases in the present study, one patient showed no recurrence within a seven-year follow-up, one patient died three years after the surgery, and the other two patients are five and six months into the postoperative follow-up period, respectively.

In conclusion, the clinicopathological features of four PEH cases were investigated and the associated literature was reviewed. This revealed PEH to be a rare yet diverse form of malignant vasular tumor with varying patient prognoses. The results of this study may improve our understanding of PEH. However, studies with a larger sample size are required in order to provide a more comprehensive understanding, which would improve the clinical treatment and prognosis for patients with PEH.

## Figures and Tables

**Table I:** Clinical data of the four cases of pulmonary epithelioid hemangioendothelioma.

Case no.	Gender	Age (years)	Clinical symptoms	Disease site	Imaging findings	Tumor diameter (cm)	Treatment	Follow-up
1	Male	54	No obvious symptom	Left lung	Multiple nodules in both lungs	4 nodules: 0.5–1.0	Wedge resection of left upper lobe and left lower lobe	7 years, in stable condition
2	Female	54	Pleural effusion on right side for 1 month	Right upper lung	Irregular pleural thickening at right upper lobe	2 lesions: 3×2×1, 1×0.5×0.5	Wedge resection of right upper lobe with postoperative chemotherapy	Succumbed 3 years after surgery
3	Female	46	No obvious symptoms	Right upper lung	Irregular clumps below the pleura of the right upper lobe	Multiple lesions: 0.3–3	Right upper lobectomy, nodular resection in right middle lower lobe and pleural nodules	Postoperative follow-up for 6 months
4	Female	30	No obvious symptoms	Left lower lung	Multiple nodules in both lungs	2 nodules: 1.5–3	Left lower lobectomy	Postoperative follow-up for 5 months
